# Evidence that high von Willebrand factor and low ADAMTS-13 levels independently increase the risk of a non-fatal heart attack

**DOI:** 10.1111/j.1538-7836.2008.02902.x

**Published:** 2008-04-01

**Authors:** J T B CRAWLEY, D A LANE, M WOODWARD, A RUMLEY, G D O LOWE

**Affiliations:** * Department of Haematology, Imperial College London London, UK; † Department of Medicine, Mount Sinai Medical Center New York, NY, USA; ‡ Division of Cardiovascular and Medical Sciences, University of Glasgow Glasgow, UK

**Keywords:** ADAMTS-13, Glasgow Myocardial Infarction Study, myocardial infarction, VWF

## Abstract

*Background:* A disintegrin and metalloproteinase with a thrombospondin type 1 motif, member 13 (ADAMTS-13) may influence von Willebrand factor (VWF) levels and consequently the risk of myocardial infarction (MI). Moreover, ADAMTS-13 influences hemostatic plug formation in mouse models. We therefore studied their associations in the Glasgow MI Study (GLAMIS). *Methods and results:* We measured ADAMTS-13 and VWF antigen levels by ELISAs in stored plasma from a case–control study of 466 MI cases and 484 age- and sex-matched controls from the same north Glasgow population. There was no correlation between ADAMTS-13 and VWF levels in cases or controls. ADAMTS-13 levels correlated positively with serum cholesterol and triglycerides and body mass index, and negatively with high-density lipoprotein-cholesterol. VWF levels correlated with age, fibrinogen and C-reactive protein. In multivariable analyses including risk factors, VWF correlated positively with risk of MI, and ADAMTS-13 correlated negatively with risk of MI. These associations were independent of each other. The association of ADAMTS-13 with risk of MI was observed only in multivariable analysis. *Conclusions:* VWF and ADAMTS-13 levels were not associated in this study, and showed associations with MI risk in opposite directions but of similar strength. The association of ADAMTS-13 with MI is influenced by lipid levels, and consequently requires further investigation.

## Introduction

Von Willebrand factor (VWF) plays an important role in hemostasis and thrombosis, both as a cofactor in platelet adhesion and aggregation and as the circulating carrier protein for coagulation factor VIII [1]. Meta-analyses of prospective studies have suggested that increasing circulating VWF levels are associated with increased risk of coronary heart disease (CHD) [2,3], although in the most recently reported meta-analysis this association was relatively weak [3]. The molecular weight/multimeric composition of VWF is a key determinant of its platelet-tethering function. This is modulated by a disintegrin and metalloproteinase with a thrombospondin type 1 motif, member 13 (ADAMTS-13). ADAMTS-13 cleaves the VWF A2 domain, reducing its molecular weight and consequently also its platelet-tethering function [4–8]. Deficiency of ADAMTS-13 promotes VWF-induced platelet aggregation, which can result in thrombotic thrombocytopenic purpura [4]. In mouse models of thrombosis, ADAMTS-13 downregulates both platelet adhesion to the exposed subendothelial matrix and thrombus formation in injured arterioles [9]. It has therefore been suggested that circulating levels of ADAMTS-13 may influence circulating levels of VWF and/or its function, and it may therefore influence risk of thrombotic events such as myocardial infarction (MI) in the general population.

We have studied this hypothesis in a case–control study of first episode of MI, the Glasgow Myocardial Infarction Study (GLAMIS) [10]. We studied the distributions and correlations of VWF and ADAMTS-13 with CHD risk factors, and with each other. Thereafter, we studied the associations of VWF and ADAMTS-13 with MI, following adjustment for risk factors – including high-density lipoprotein (HDL)-cholesterol, fibrinogen and C-reactive protein (CRP) [10] – and each other.

## Subjects and methods

GLAMIS [10] is a case–control study wherein cases of MI were derived from the international MONICA study [11] register in north Glasgow [12] in 1994–1995. Survivors on the register were recruited between 3 and 9 months after their MI event. We have previously studied VWF serially between 1 and 6 months following MI values. These data showed that VWF levels were stable and lower than on admission [13]. Consequently, the sampling of plasmas between 3 and 9 months post-MI event in this study should not be influenced by the acute-phase protein response to MI. The time lag between the event and recruitment ensured that acute-phase protein reactions in acute MI had settled. Controls were selected from a random sample of the same north Glasgow population, obtained from general practice registers. Controls were frequency-matched for sex (726 men and 269 women) and age (within 1 year), and had no self-reported history, or electrocardiogram evidence, of MI. In all but 2.5% of controls, electrocardiography verified the absence of evidence of MI. Written informed consent was obtained from all participants, and the study was approved by the local Research Ethics Committee.

Participants completed a general health questionnaire (taken after the MI in the case of patients), which included questions on age (range 25–64 years), sex, diabetes status (yes/no), smoking status (current smoker/ex-smoker/never smoked cigarettes), and medication used, which related to the time after the MI in the case of patients. Subjects attended at a clinic where weight, height and blood pressure were recorded. Body mass index (BMI) was calculated as weight divided by the square of height. A forearm venous sample was taken after a full overnight fast. Lipid assays were measured as previously described [9]. The assays of inflammatory and hemostatic variables used blood anticoagulated with dipotassium ethylenediaminetetraacetic acid (EDTA; 1.5 mg mL^−1^), centrifuged at 2000 *g* for 10 min at room temperature within 2 h of sampling, and aliquots were stored at −70°C. Fibrinogen was assayed in citrated plasma using the von Clauss method on an MDA-180 analyzer (Organon Teknika, Cambridge, UK) and an ultra-sensitive assay for CRP was performed in citrated plasma on a nephelometer (Dade-Behring, Marburg, Germany), as previously described [10]. VWF antigen was assayed in citrated plasma by an in-house ELISA, using reagents from DAKO (Copenhagen, Denmark); the interassay coefficient of variance (CV) was 3.3%. ADAMTS-13 was assayed in dipotassium EDTA plasma using an in-house ELISA as previously described [14] with an interassay CV of <5%.

### Statistical analysis

VWF, ADAMTS-13, continuous major cardiovascular risk factors (age, blood pressure, serum lipids, BMI), fibrinogen and CRP were compared between MI cases and controls using general linear models, which adjusted for age and sex. Skewed continuous variables were transformed; VWF and ADAMTS-13 were approximately normally distributed after a log and a square root transformation, respectively. Binary major cardiovascular risk factors (whether the subject was: male; had diabetes; had ever smoked; was currently taking medication for high blood pressure; or was currently taking medication for high cholesterol) were compared between MI cases and controls using logistic regression models, which adjusted for age and sex. The age- and sex-adjusted relationships between both VWF and ADAMTS-13 and each of the continuous potential confounding variables, taken one at a time, were estimated for controls from general linear models, after transforming each of the dependent variables to approximate normality. Age- and sex-adjusted correlations between VWF and ADAMTS-13 were also estimated after these transformations. Logistic regression models were used to obtain odds ratios (ORs) for MI by equal thirds of the values for VWF and ADAMTS-13 (in both patients and controls) and for a continuous 1 standard deviation (SD) increase in each variable amongst the controls. Three sets of adjustments were used in evaluating the association between VWF and the risk of MI, each with and without further adjustment for ADAMTS-13: age and sex; all continuous and binary major cardiovascular risk factors; and additionally fibrinogen and CRP. Similar adjustments were made for ADAMTS-13, with and without adjustment for VWF. Interactions were tested by adding terms to the relevant statistical models [15].

## Results

ADAMTS-13 or VWF data were available for 950 plasma samples. Due to incomplete residual aliquots, VWF was measured in 875 (88%) and ADAMTS-13 in 919 (94%) of the 995 participants in GLAMIS. Cases had significantly higher average values for triglycerides, BMI, fibrinogen, CRP and VWF than controls, but lower values for blood pressure and HDL-cholesterol (Table 1). Cases were also more likely than controls to have ever smoked (89% vs. 74%), to have diabetes (12% vs. 2%), and to be taking medication for high blood pressure (39% vs. 17%) or for high cholesterol (28% vs. 1%) (data not shown). As anticipated by design, there were no significant differences in either age or sex between cases and controls. There were also no significant differences in levels of total cholesterol or ADAMTS-13.

**Table 1:** Summary statistics for ADAMTS-13, von Willebrand factor (VWF) and continuous coronary risk factors

	Controls (*n* = 484)						Cases (*n* = 466)						
		
	n	Mean	SD	Median	1st Quartile	3rd Quartile	n	Mean	SD	Median	1st Quartile	3rd Quartile	*P*-value*
Age (years)	484	55.1	7.45	57	50	61	466	54.9	7.46	57	51	61	0.76
Systolic BP(mmHg)	484	130	19.9	130	116	144	465	123	21.8	121	105	137	<0.0001
Diastolic BP(mmHg)	484	82.8	11.1	83	75	90	465	78.4	12.8	77	69	87	<0.0001
Total cholesterol(mmol L^−1^)	478	5.76	1.16	5.7	4.9	6.5	463	5.80	1.16	5.7	4.9	6.5	0.55
HDL-cholesterol(mmol L^−1^)	477	1.35	0.42	1.3	1.0	1.6	463	1.12	0.31	1.1	0.9	1.3	<0.0001
Triglycerides(mmol L^−1^)	478	2.10	6.98	1.5	1.0	2.1	463	2.21	1.38	1.9	1.4	2.6	<0.0001
BMI (kg m^−2^)	484	26. 9	4.62	26	23	30	463	27.9	4.80	27	24	30	0.0003
Fibrinogen (g L^−1^)	378	3.12	0.67	3.0	2.7	3.5	381	3.44	0.75	3.4	2.9	3.9	<0.0001
CRP (mg L^−1^)	370	4.17	8.56	1.8	0.8	4.2	385	5.26	6.94	2.9	1.3	6.3	<0.0001
ADAMTS-13 (%)	472	112.6	32.6	106	92	127	447	111	35.9	105	88	125	0.36
VWF (IU dL^−1^)	414	164	60.3	154	121	199	461	186	68.1	178	141	219	<0.0001

BP, blood pressure.

* Test of equality of means, cases vs. controls, after adjustment for age and sex and log transformation for high-density lipoprotein (HDL)-cholesterol, triglycerides, body mass index (BMI), fibrinogen, C-reactive protein (CRP) and VWF and square root transformation for ADAMTS-13. Age was adjusted for sex.

In Table 2, plasma levels of ADAMTS-13 and VWF in control subjects (for whom both values were available) are shown according to the different binary variables. In controls there was no significant age-/sex-adjusted difference (*P* > 0.05) in mean VWF by sex, ever-smoking status, diabetes status, or use of medication for high blood pressure or for high cholesterol (Table 2). ADAMTS-13 was higher in those taking compared with those not taking medication for high blood pressure. None of the other comparisons of ADAMTS-13 levels showed any differences amongst the different groups analyzed.

**Table 2:** Plasma levels of ADAMTS-13 and von Willebrand factor (VWF) in controls amongst the different binary variables examined [i.e. according to sex, smoking, diabetes, and the taking of medication for high blood pressure (BP) or high cholesterol]. *P*-values after transformations as in Table 1

	Yes						No						
		
	n	Mean	SD	Median	1st Quartile	3rd Quartile	n	Mean	SD	Median	1st Quartile	3rd Quartile	*P*-value*
Male? ADAMTS-13	296	111	32.5	107	89.3	126	106	114	34.6	106	93.8	120	0.27
VWF	296	165	62.1	156	121	198	106	160	54.9	152	120	200	0.16
Ever smoked? ADAMTS-13	302	110	32.5	106	88.9	125	98	118	34.3	108	96.0	129	0.11
VWF	302	166	61.4	157	119	201	98	157	56.6	144.5	122	189	0.60
Diabetes? ADAMTS-13	7	126	30.8	117	100	145	393	112	33.1	106	90.8	126	0.20
VWF	7	192	66.3	195	133	261	393	163	60.2	154	121	197	0.32
High BP medication?ADAMTS13	66	123	40.5	112	98.4	140	334	110	31.0	105.2	90.3	125	0.0009
VWF	66	174	65.6	175	130	207	334	161	59.1	152	120	196	0.44
High cholesterolmedication? ADAMTS-13	5	124	32.0	127	102	129	395	112	33.1	106	90.8	126	0.37
VWF	5	212	95.7	176	175	198	395	163	59.7	154	120	197	0.15

Age-/sex-adjusted correlations between transformed values of ADAMTS-13 and VWF were −0.01 (*P* = 0.81) for controls and −0.06 (*P* = 0.23) for cases (data not shown). From analysis of control individuals for whom both ADAMTS-13 and VWF values were available (*n* = 484), there were positive relationships between VWF and age and fibrinogen (Table 3). Similarly, there were strong positive correlations between ADAMTS-13 and total cholesterol, triglycerides and BMI (Table 3). There was also a notable negative correlation between ADAMTS-13 and HDL-cholesterol.

**Table 3:** Age- and sex-adjusted relationships between ADAMTS-13, VWF and continuous coronary risk factors amongst controls. General linear models were fitted with transformed VWF or ADAMTS-13 as dependent variables (transformations as in Table 1). Beta coefficients associated with each factor are shown with the *P*-value for test of no association

	Controls	
	
	ADAMTS-13	VWF
Age*	−0.019 (*P* = 0.04)	0.010 (*P* < 0.0001)
Systolic BP	0.0003 (*P* = 0.94)	0.0003 (*P* = 0.76)
Diastolic BP	0.006 (*P* = 0.32)	−0.001 (*P* = 0.36)
Total cholesterol	0.205 (*P* = 0.0005)	0.017 (*P* = 0.28)
HDL-cholesterol	−0.678 (*P* < 0.0001)	−0.016 (*P* = 0.71)
Triglycerides	0.021 (*P* = 0.03)	0.009 (*P* = 0.22)
BMI	0.077 (*P* < 0.0001)	0.004 (*P* = 0.30)
Fibrinogen	0.181 (*P* = 0.12)	0.107 (*P* = 0.0004)
CRP	0.005 (*P* = 0.57)	0.004 (*P* = 0.10)

BMI, body mass index; BP, blood pressure; CRP, C-reactive protein; HDL-cholesterol, high-density lipoprotein-cholesterol.

* Adjusted for sex.

The OR of MI increased with increasing thirds of VWF. After adjustment for age and sex, those in the highest third had about three times the risk of those in the first (Table 4). Assuming a log-linear decline is an acceptable model, an increase of 1 SD (60.3 IU dL^−1^) of VWF was estimated to increase the odds of MI by 51%, after correction for age and sex (Table 4, Adjusted I). Adjustment for ADAMTS-13 had little effect on the ORs for VWF. Adjustment for established cardiovascular disease risk factors moderately reduced the OR for VWF (Table 4, Adjusted II and III). There was no evidence of a difference in the OR for MI over increasing thirds of ADAMTS-13, after adjustment for age and sex (Table 4, Adjusted I). However, after adjustment for major cardiovascular disease risk factors, there was a clear inverse relationship between ADAMTS-13 and MI (Table 4, Adjusted II): those in the highest third had about 54% of the risk of those in the first third, whilst an increase of 1 SD (32.6%) in ADAMTS-13 was estimated to lead to a 27% reduction in the odds of MI. The OR for an increase of 1 SD in ADAMTS-13 changed from 1.01 to 0.72 after adjusting for a host of other potential confounding variables on top of age and sex. However, no single variable explained the majority of this decrease; adjustment for HDL-cholesterol caused the largest change, to 0.91. Adjustment for all the lipid variables (total cholesterol and HDL-cholesterol, triglycerides and medication for high cholesterol), as well as age and sex, resulted in an OR [95% confidence interval (95% CI)] of 0.83 (0.70–0.99; results not shown). Further adjustment for fibrinogen and CRP (‘full adjustment’; Table 4, Adjusted III) did not materially alter the effect of ADAMTS-13. Adjustment of VWF for ADAMTS-13 had no appreciable effect. There was no interaction between ADAMTS-13 and VWF in predicting MI for any of the three adjustment sets, for analyses by thirds or as continuous variables (*P*≥ 0.19).

**Table 4:** Odds ratios (95% confidence intervals) for risk of myocardial infarction for ADAMTS-13 and von Willebrand factor (VWF), adjusted for three sets of risk factors (I-III), with and without adjustment (+/−Adj.) for each other. Analyses are by thirds (first third=1) and for a continuous increase of 1 standard deviation (SD). Only subjects with no missing values of any variables are included (*n* = 669).

	ADAMTS-13		VWF	
Third	−Adj. VWF	+Adj. VWF	−Adj. ADAMTS-13	+Adj. ADAMTS-13
Adjusted I				
1	1	1	1	1
2	1.03 (0.71–1.50)	1.01 (0.69–1.48)	2.17 (1.48–3.17)	2.17 (1.49–3.18)
3	1.13 (0.78–1.64)	1.16 (0.79–1.71)	2.98 (2.02–4.38)	3.00 (2.04–4.42)
1 SD	1.01 (0.87–1.17)	1.03 (0.89–1.20)	1.50 (1.28–1.76)	1.51 (1.28–1.76)
Adjusted II				
1	1	1	1	1
2	0.72 (0.45–1.16)	0.71 (0.44–1.15)	1.80 (1.11–2.91)	1.76 (1.08–2.86)
3	0.52 (0.31–0.85)	0.54 (0.32–0.89)	2.56 (1.58–4.16)	2.51 (1.54–4.09)
1 SD	0.72 (0.58–0.88)	0.73 (0.59–0.90)	1.42 (1.18–1.71)	1.40 (1.16–1.68)
Adjusted III				
1	1	1	1	1
2	0.76 (0.47–1.23)	0.74 (0.45–1.20)	1.69 (1.04–2.75)	1.66 (1.01–2.71)
3	0.51 (0.31–0.85)	0.53 (0.32–0.88)	2.33 (1.42–3.83)	2.28 (1.38–3.75)
1 SD	0.72 (0.58–0.88)	0.73 (0.59–0.90)	1.37 (1.13–1.66)	1.35 (1.11–1.63)

Adjusted I: age and sex. Adjusted II: age, sex, systolic blood pressure (BP), diastolic BP, total serum cholesterol, high-density lipoprotein (HDL)-cholesterol, triglycerides, diabetes, smoking (current/ex-/never), body mass index (BMI), use of medications for high BP and high cholesterol. Adjusted III: age, sex, systolic BP, diastolic BP, total serum cholesterol, HDL-cholesterol, triglycerides, diabetes, smoking (current/ex-/never), BMI, use of medications for high BP and high cholesterol, fibrinogen and C-reactive protein.

Notes: The tertiles for ADAMTS-13 are 95.8 and 117.7% and for VWF are 144 and 195I U dL^−1^. One SD is given (for controls) in Table 1.

When ADAMTS-13 and VWF were varied together, the OR (95% CI) comparing those in the highest third of VWF and the lowest third of ADAMTS-13 with those in the lowest third of VWF and the highest third of ADAMTS-13, after full adjustment, was 4.70 (1.76–12.6). This OR is very similar to the OR of 4.57 that was obtained by multiplication of the fully adjusted ORs contrasting the extreme thirds when ADAMTS-13 and VWF were analyzed independently of the other, restricting analysis to subjects with data on both variables. This underlines the independence of their effects on MI.

## Discussion

This study suggests that both ADAMTS-13 and VWF antigen are risk factors for a major non-fatal coronary event (MI), with effects that are in opposite directions, and are clearly independent of each other. VWF has effects that are largely unaffected by other cardiovascular disease risk factors, unlike ADAMTS-13, which appears to have effects that are masked by the coexistence of other risk factors. After adjustment for the largest set of cardiovascular risk factors considered here, an additional 1 SD of VWF (about 60 IU dL^−1^) is expected to raise the risk of MI by about 35%, similar to the amount (27%) by which the risk is lowered after increasing ADAMTS-13 by 1 SD. The association of VWF antigen with MI in the current case–control study is consistent with a recent meta-analysis of prospective studies of CHD [3].

Our finding of a lack of association between plasma levels of VWF and ADAMTS-13 is in agreement with another recent case–control study of similar size [14]; hence, it appears unlikely that ADAMTS-13 appreciably affects VWF antigen levels across the population range. A previous study reported an inverse relationship between VWF and ADAMTS-13 [16]; however, these data revealed a moderate inverse correlation in a small number of individuals and reported a more marked effect following acute release of VWF. In that study ADAMTS-13 activity assays were used to measure ADAMTS-13 levels [16]. It remains uncertain how VWF levels in the plasma samples may or may not have influenced the activity data obtained. Although the study found an inverse relationship between plasma VWF and ADAMTS-13 activity levels measured following the acute-phase response to desmopressin, the temporal changes in ADAMTS-13 and VWF levels are open to several explanations. It may be beneficial to measure both ADAMTS-13 antigen and activity levels in order to more accurately assess patient samples in future studies. Unfortunately, determining ADAMTS-13 activity in the plasma samples analyzed herein was not possible as the plasmas were anticoagulated with EDTA, which precludes functional analysis of ADAMTS-13. That aside, accurate determination of ADAMTS-13 activity data can be impractical for large patient populations due to the difficulties/variability and costs associated with such assays.

The important finding that distinguishes the present study from our previous case–control study is that in that study [14] we found an unexpected increase in risk of MI with increasing ADAMTS-13, whereas the present study found a decrease in risk, albeit only after adjusting for CHD risk factors, which were not reported in the earlier study [14]. The earlier study comprised only male MI patients and controls, whereas the present study investigated both men and women; however, even when we restricted our analysis to males, the results were almost identical (data not shown), suggesting that this was not the cause of the conflicting data. There were differences in the age ranges of the two study populations but controlling for age did not appear to alter the findings of the two studies (not shown).

Of the risk factors that we adjusted for, by far the most important in masking the true association between ADAMTS-13 and MI was HDL-cholesterol. Future studies of ADAMTS-13 may therefore need to take account of lipids, including HDL-cholesterol, when assessing its association with MI. However, at this time there is no mechanistic/biochemical data that might explain this observed association. It must also be considered that in epidemiology it is unusual to find a confounding effect where one variable's effect only appears after adjustment for another. Most often, adjustment attenuates an effect. The unusual situation here is that the independent effects of HDL-cholesterol and ADAMTS-13 both act in the same (negative) direction on the risk of MI, yet the two variables are negatively correlated with each other. Our results suggest that it may be interesting to investigate the effect of plasma lipids on ADAMTS-13.

The data from our present study in part agree with two separate studies from the same group of investigators who recently found that an increase in the VWF:ADAMTS-13 ratio was associated with subsequent cardiovascular events in hospital post-MI in a small number of patients [17,18]. However, it must be considered that, unlike in our case–control studies, these data analyzed VWF and ADAMTS-13 at time of admission to hospital (and in one study over the subsequent 14 days), rather than after the end of the acute-phase response. This is an important consideration when comparing these data sets.

The present case–control study has the disadvantage of using a study design that is relatively weak for estimating causality [15]. In particular, survivor bias may have had some effect on the results (cases had to be alive several months after their MI). The lag time between the event and recruitment into the study, for cases, was ideal for ensuring that transient inflammatory changes, consequent to the event, had disappeared, but did leave time for changes to lifestyle and treatment that will usually occur after surviving a significant medical event. This is manifested here by the lower blood pressures in the case group, the lack of difference between the cases and controls in average cholesterol levels, and the same percentage (45%) of current smokers amongst cases and controls at the time of surveying. To deal with this problem, correlations and binary relationships with ADAMTS-13 and VWF were analyzed separately for cases and controls (although only the latter are presented); medication use is included as an adjustment variable in the analyses of association with MI; and smoking status is dichotomized as ever or never smoked cigarettes. Another limitation might be the poor general health of the north Glasgow control group used here, reflected by the high prevalence of smoking and taking blood pressure-lowering treatment, and the suboptimal average values of risk factors such as BMI. This might be expected to have led to an underestimation of the effects of the associations between the two novel risk factors considered here and MI.

We observed positive associations of VWF with fibrinogen, consistent with the known acute-phase reactant behavior of VWF. We also observed negative associations of ADAMTS-13 with fibrinogen and CRP, but only in cases (data not shown). As in the previous study [14], there was little/no effect of adjustment for fibrinogen and CRP on the associations between VWF or ADAMTS-13 and risk of MI.

In conclusion, we report that VWF and ADAMTS-13 levels were not associated in this case–control study of MI; that ADAMTS-13 levels were associated with blood lipid levels (cholesterol, triglyceride, HDL-cholesterol) and obesity (BMI); that VWF and ADAMTS-13 levels showed respectively positive and negative associations with risk of MI; and that the latter association was apparent only after adjustment for CHD risk factors, in particular HDL-cholesterol. Further studies, including adjustment for effects of blood lipids and measurement of VWF multimers, are suggested in order to clarify the relationships between ADAMTS-13, VWF and cardiovascular risk.
